# Neuroprotective Properties of Quinone Reductase 2 Inhibitor M-11, a 2-Mercaptobenzimidazole Derivative

**DOI:** 10.3390/ijms222313061

**Published:** 2021-12-02

**Authors:** Mikhail V. Voronin, Ilya A. Kadnikov, Liana F. Zainullina, Ilya O. Logvinov, Ekaterina R. Verbovaya, Tatyana A. Antipova, Yulia V. Vakhitova, Sergei B. Seredenin

**Affiliations:** Department of Pharmacogenetics, Federal State Budgetary Institution “Research Zakusov Institute of Pharmacology”, Baltiyskaya Street 8, 125315 Moscow, Russia; zainullinalf@gmail.com (L.F.Z.); logvinov_ilya@mail.ru (I.O.L.); zueva.kate1997@gmail.com (E.R.V.); zenina_tatyana@mail.ru (T.A.A.)

**Keywords:** NQO2, adrenochrome, BNAH, ROS, comet assay, apoptosis, HT-22 cells, NQO2 inhibitors, 2-mercaptobenzimidazole derivative, S29434

## Abstract

The ability of NQO2 to increase the production of free radicals under enhanced generation of quinone derivatives of catecholamines is considered to be a component of neurodegenerative disease pathogenesis. The present study aimed to investigate the neuroprotective mechanisms of original NQO2 inhibitor M-11 (2-[2-(3-oxomorpholin-4-il)-ethylthio]-5-ethoxybenzimidazole hydrochloride) in a cellular damage model using NQO2 endogenous substrate adrenochrome (125 µM) and co-substrate BNAH (100 µM). The effects of M-11 (10–100 µM) on the reactive oxygen species (ROS) generation, apoptosis and lesion of nuclear DNA were evaluated using flow cytometry and single-cell gel electrophoresis assay (comet assay). Results were compared with S29434, the reference inhibitor of NQO2. It was found that treatment of HT-22 cells with M-11 results in a decline of ROS production triggered by incubation of cells with NQO2 substrate and co-substrate. Pre-incubation of HT-22 cells with compounds M-11 or S29434 results in a decrease of DNA damage and late apoptotic cell percentage reduction. The obtained results provide a rationale for further development of the M-11 compound as a potential neuroprotective agent.

## 1. Introduction

Epidemiological data indicate an increasing prevalence of neurodegenerative diseases, especially in countries with high life expectancy [1]. Pathogenesis of Alzheimer’s, Parkinson’s and Huntington’s diseases include imbalance of cellular bioenergetics, increased ROS production, protein misfolding resulting in neuron and glial cells loss, synaptic deterioration and decreased neuroplasticity [2,3,4], which determine physiological function impairment and clinical symptoms [5]. Oxidative stress is considered to be the main mechanism damaging pivotal cell macromolecules [6,7,8,9]. Numerous reviews and monographs have described causes and sources of overproduction of free radicals [10,11,12]. Extensive information on natural and synthesized antioxidant use for central nervous system disease treatment has been accumulated [13,14]. However, their pharmacotherapeutic use requires further elaboration based on the study of pathogenetic mechanisms [15,16].

Disturbances in the metabolism of catecholamines with excessive production of their quinone derivatives and reactive oxygen species (ROS) provide a significant contribution to the oxidative stress [17,18,19,20,21,22,23,24]. For example, dopamine, a precursor of other neurotransmitter catecholamines, after being synthesized in the cytosol, is transported to synaptic vesicles by the synaptic vesicular amine transporter (VAT2; VMAT2) with the participation of V-type proton ATPase (V-ATPase) and glyceraldehyde-3-phosphate dehydrogenase (GAPDH) [25,26]. V-ATPase-dependent proton gradient provides a pH of ~5.6 in synaptic vesicles which ensures stability of catecholamines [27]. However, in neurodegenerative diseases, oxidative modification of GAPDH [28] mediates impairments to the functional activity of VAT2 [29], leading to a decreased uptake of catecholamines to vesicles both after presynaptic reuptake or de novo synthesis [30,31] and their autoxidation to quinones at pH 7.4 [27,32]. Quinone derivatives of catecholamines, possessing electrophilic properties, form conjugates with NH2-groups of amino acid residues of proteins and nucleic acid bases, disrupting their native conformation and functional activity [33,34,35].

**Figure 1:**
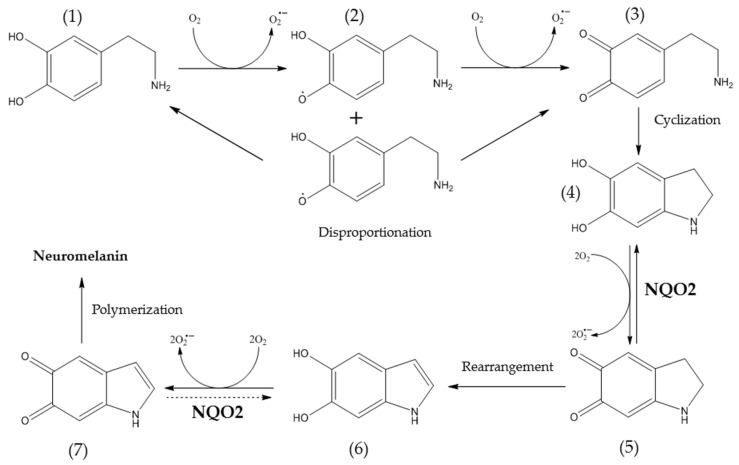
Oxidation of dopamine as a possible source of NQO2 endogenous substrates. The scheme is based on Segura-Aguilar et al. [42] and Cassagnes et al. [44]. (**1**)–dopamine, (**2**)–dopamine semiquinone, (**3**)–dopamine o-quinone, (**4**)–leukoaminochrome, (**5**)–aminochrome, (**6**)–5,6-dihydroxyindole, (**7**)–5,6-indolequinone. Synthesis of (**3**), (**5**) and (**7**) passes through formation of respective semiquinones that may undergo disproportionation reaction when two identical semiquinones interact, generating respective quinone and hydroquinone. A dashed arrow corresponds to the putative reaction that is catalyzed by NQO2.

Flavin-dependent enzyme ribosyldihydronicotinamide dehydrogenase [quinone] (NQO2; QR2) is involved in the detoxification of quinone derivatives of catecholamines, catalyzing a two-electron reduction of para-quinones (1,4-quinones), ortho-quinones (1,2-quinones) and pseudoquinones [36,37]. However, the quinols (hydroquinones) formed during the enzymatic reaction undergo autooxidation to semiquinones [36,38,39], which are capable of transferring their unpaired electron to other oxidants, including molecular oxygen [40] (Figure 1). The reaction produces superoxide anion radicals (O_2_^•−^) and the corresponding quinone [38,39,40,41]. In addition, semiquinones can interact with each other to form quinone and initial quinol [42]. Reduction of quinone, catalyzed by NQ_O2_, renews the redox cycle [43] (Figure 1).

Thus, in neurodegenerative diseases under conditions of insufficient antioxidant systems [45,46], these reactions lead to an increase in the production of free radicals. Therefore, it can be assumed that a therapeutic effect is achieved due to a decrease in NQO2 activity [39,47,48].

NQO2 is expressed in different regions of the human brain [49,50] and is predominantly localized in cell cytosol [36]; the existence of NQO2 in nucleoplasm was also observed [50,51]. Human NQO2 consists of 231 amino acid residues (26 kDa) [52] and includes catalytic (1–220 aa) and C-terminal domains (221–231 aa) [53]. After cloning the NQO2 gene, determining the amino acid sequence of the enzyme [52], as well as analysis of ligand binding [54,55] revealed the NQO2 regulatory site to be designated as an MT_3_ receptor [56]. Known ligands of the MT_3_ receptor are melatonin and its derivatives [57], benzimidazole derivatives TBB, TBBz, DMAT [58], flavonoids resveratrol [59] and quercetin [60], α-adrenergic receptor antagonist prazosin [61] inhibit NQO2 [36] and possess neuroprotective properties. However, these compounds are not selective ligands of the MT_3_ receptor, and its mechanisms of neuroprotective action include other molecular targets [62,63,64,65,66].

**Figure A1:**
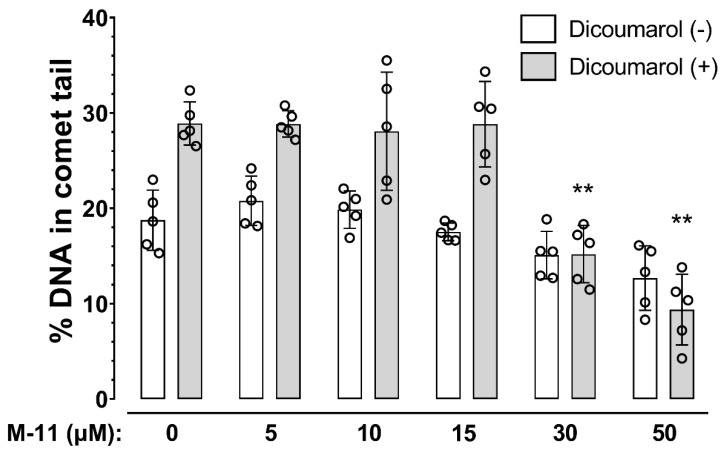
The influence of M-11 on nuclear DNA damage of ICR mice bone marrow cell suspension caused by menadione. Suspension of bone marrow cells of ICR mice was incubated with M-11 (5–50 µM) in presence or absence of dicoumarol (10 µM) for 30 min. After pre-incubation, cells were incubated with menadione (25 µM) for 60 min. Data are presented as mean ± S.D. Two-way ANOVA with Tukey’s post-hoc was used: ** *p* < 0.001 vs. pre-incubation without M-11.

During pharmacokinetic studies of anxiolytic afobazole (ethoxy-2-[2-(morpholino)-ethylthio]benzimidazole dihydrochloride) [67], interacting with chaperone Sigma1R (Ki = 5.9 μM), NQO2 (MT_3_ receptor, Ki = 0.97 μM) and monoamine oxidase A (MAO-A, Ki = 3.6 μM) regulatory sites [68], carried out at the FSBI “Zakusov Institute of Pharmacology”, a metabolite of afobazole 2-[2-(3-oxomorpholine-4-yl)ethylthio]-5-ethoxybenzimidazole hydrochloride (M-11) was found [69,70]. Radioligand analysis (Eurofins Cerep) have indicated that compound M-11 interacts only with the NQO2 regulatory site (Ki = 0,39 μM) [68]. We have shown that M-11 inhibits NQO2 [71], and possesses cytoprotective [72,73] and neuroprotective [74] effects. Our experiments did not reveal any influence of M-11 on the effects related to the activity of homologous enzyme NQO1 (Figure A1) [72,75].

Single-cell gel electrophoresis assay, also known as comet assay, allows one to evaluate nuclear DNA damage as a consequence of oxidative stress [76,77]. An increase in the amount of DNA in the comet tail reflects the generation of alkali-labile sites (apurinic/apyrimidinic sites) and DNA strand breaks [78]. However, morphologically atypical comets (hedgehog comets, cloud comets, ghost cells, high damage cells) can be observed and, generally, this type of comet is not taken into account during analysis. It should be noted that oxidative stress is considered as one of mechanisms contributing to the formation of atypical comets, which was demonstrated in the presence of high concentrations of H_2_O_2_ [79,80].

In this study, we took advantage of the in vitro experimental model, by using the endogenous NQO2 substrate adrenochrome and co-substrate BNAH to evaluate the mechanisms of neuroprotective activity of M-11 based on analysis of ROS-triggered apoptosis and oxidative nuclear DNA damage assessed by comet assay. The effects of M-11 were compared with S29434, a known NQO2 inhibitor [81].

## 2. Results

**Figure 2:**
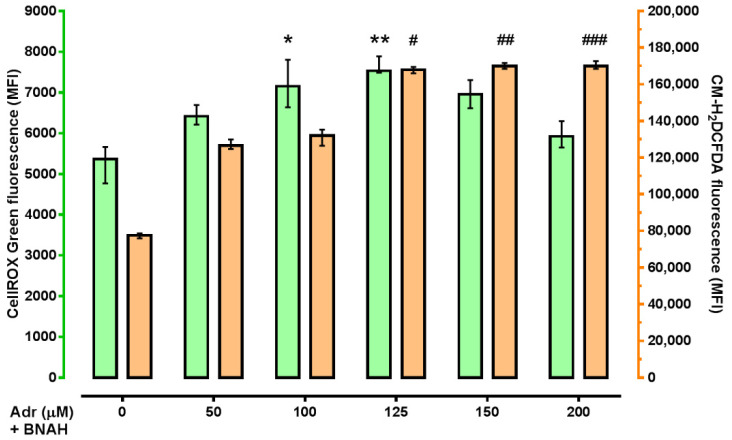
The effect of adrenochrome and BNAH on ROS production in HT-22 cells. Cells were incubated with adrenochrome (50–200 µM) and/or BNAH (100 µM) for 15 min. Final concentration of DMSO was 0.2%. Data are presented as median (min–max). All experiments were performed in 12 replicates. Kruskal–Wallis test with Dunn’s post-hoc: * *p* < 0.05, ** *p* < 0.01 vs. Adrenochrome (0 μM) + BNAH group in ROS measurement with CellROX Green Reagent; # *p* < 0.05, ## *p* = 0.003, ### *p* < 0.001 vs. Adrenochrome (0 μM) + BNAH group in ROS measurement with CM-H_2_DCFDA. For details see Table S1.

We used HT-22 cells treated with NQO2 endogenous substrate adrenochrome and co-substrate BNAH as an in vitro model for NQO2-mediated ROS production, since HT-22 cells express NQO2 [82] (Figure S1) and NQO2 has been shown to be capable of producing ROS in reaction with the aforementioned substrate and co-substrate [43]. Incubation of HT-22 cells with adrenochrome (50–200 µM concentration range) and BNAH (100 µM) causes a statistically significant increase in the CellROX Green signal for sub-cellular superoxide (O_2_^•−^) with maximum fluorescence at 125 µM of adrenochrome (Figure 2) compared to control cells («adrenochrome (0 μM) + BNAH group»). General ROS levels detected using CM-H_2_DCFDA dye, showed an increase in a concentration-dependent manner with the maximum of fluorescence values at 125–200 µM of adrenochrome (Figure 2). Thus, a 125 µM concentration of adrenochrome has proven to be the most active in these assays and was used for subsequent experiment series with studied compounds.

**Figure 3:**
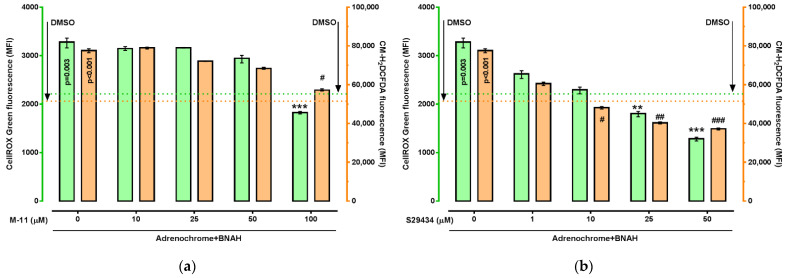
The effect of NQO2 inhibitors on the level of ROS elicited by adrenochrome and BNAH in HT-22 cells. Cells were pre-incubated with M-11 (10-100 µM) (**a**) or S29434 (1-50 µM) (**b**) for 30 min, following incubation with a combination of adrenochrome (125 µM) and BNAH (100 µM) for 15 min. All experiments were performed in 9 replicates. Data are presented as median (min–max). Kruskal–Wallis test with Dunn’s post-hoc: ** *p* < 0.01, *** *p* < 0.001 vs. M-11 (0 μM) or S29434 (0 μM) + adrenochrome/BNAH group in ROS measurement with CellROX Green Reagent; # *p* < 0.05, ## *p* < 0.01, ### *p* < 0.001 vs. M-11 (0 μM) or S29434 (0 μM) + adrenochrome/BNAH group in ROS measurement with CM-H_2_DCFDA. *p* = 0.03, *p* < 0.001—statistical significance vs. corresponding DMSO groups. For details see Table S2.

Pre-incubation of cells for 30 min with M-11 or S29434 prevented the oxidative stress caused by 15 min incubation with adrenochrome (125 µM) and BNAH (100 µM) in a concentration-dependent manner (Figure 3). In particular, pre-treatment of cells with M-11 followed by adrenochrome + BNAH incubation led to a decrease in CellROX Green Reagent (*p* < 0.001) and CM-H_2_DCFDA (*p* < 0.05) fluorescence at 100 µM of M-11 compared to control cells («adrenochrome + BNAH group») (Figure 3a). Compound S29434 attenuated CellROX Green Reagent fluorescence at concentrations of 25 µM (*p* < 0.01) and 50 µM (*p* < 0.001), while a decrease in CM-H_2_DCFDA fluorescence was detected at concentrations of 10 µM (*p* < 0.05), 25 µM (*p* < 0.01) and 50 µM (*p* < 0.001) compared to control cells («adrenochrome + BNAH group») (Figure 3b).

These data demonstrate both an increase in ROS production upon HT-22 cells incubation with the NQO2 substrate and co-substrate, and the ability of the M-11 compound, as well as the reference NQO2 inhibitor S29434, to reduce the production of ROS.

**Figure 4:**
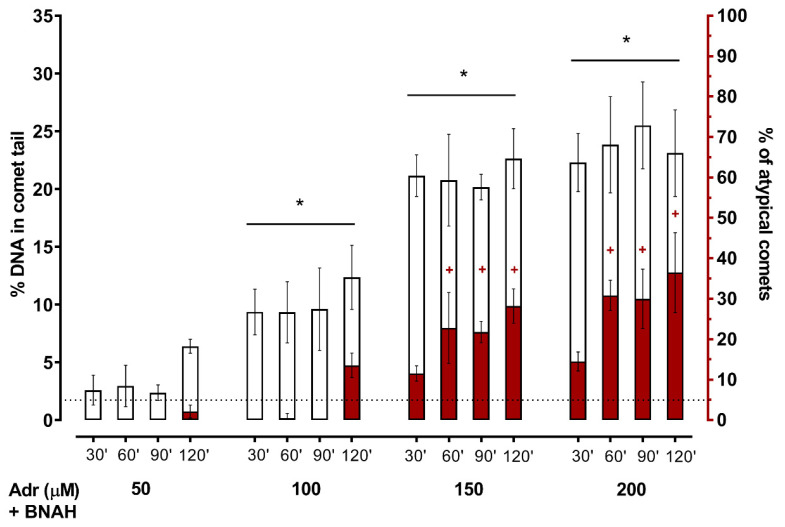
Time and concentration dependencies on DNA damage caused by adrenochrome and BNAH in HT-22 cells. Cells were incubated with adrenochrome (50–200 µM) and BNAH (100 µM) for 30, 60, 90 and 120 min. Adrenochrome and BNAH were dissolved in DMSO, final concentration of DMSO was 2%. All experiments were performed in 6 replicates. Data are presented as mean ± S.D. Data on the percentage of DNA in the comet tail (blank bars) were evaluated using one-way ANOVA with Tukey’s post-hoc: * *p* < 0.001 vs. DMSO (dotted line). Data on the percentage of atypical comets (red bars) were evaluated using Kruskal-Wallis test with Dunn’s post-hoc: + *p* < 0.05 vs. DMSO. For details see Table S3.

In the following series of experiments, the concentration and time dependences of endogenous NQO2 substrate adrenochrome effects on nuclear DNA damage of HT-22 cells were characterized (Figure 4). Adrenochrome at 100, 150 and 200 µM concentrations increased the percentage of DNA in the tail of typical comets (Figure S2) in all selected time ranges of co-incubation with BNAH (100 µM) (Figure 4). Levels of atypical DNA comets (Figure S2) increased after 60, 90 and 120 min of cell co-incubation with adrenochrome at 150 and 200 µM concentrations and BNAH (100 µM) (Figure 4).

A two-way ANOVA test revealed the dependence of incubation time with adrenochrome and BNAH (F = 8.53, *p* < 0.001) or adrenochrome concentration (F = 360.8, *p* < 0.001) on the percentage of DNA in the comet tail. At the same time, there was no relationship between these factors (F = 0.61, *p* = 0.78). Adrenochrome at 150 and 200 µM concentrations increased the percentage of DNA in the comet tail compared to the corresponding incubation time at 50 or 100 µM. An increase in adrenochrome concentration from 150 to 200 µM leads to a higher percentage of DNA in the comet tail only for 90 min incubation time (Figure 4).

A two-way ANOVA test of intergroup differences in the percentage of atypical comets was carried out for adrenochrome concentrations of 150 and 200 µM, which produced atypical comets at all incubation time points. Production of atypical comets depended on the incubation time (F = 23.26, *p* < 0.001) or adrenochrome concentration (F = 16.48, *p* < 0.001). There was no relationship between these factors (F = 0.59, *p* = 0.62). Adrenochrome (150 and 200 µM) at 60–120 min exposure caused a similar increase in the percentage of atypical comets compared to 30 min incubation times (Figure 4).

**Figure 5:**
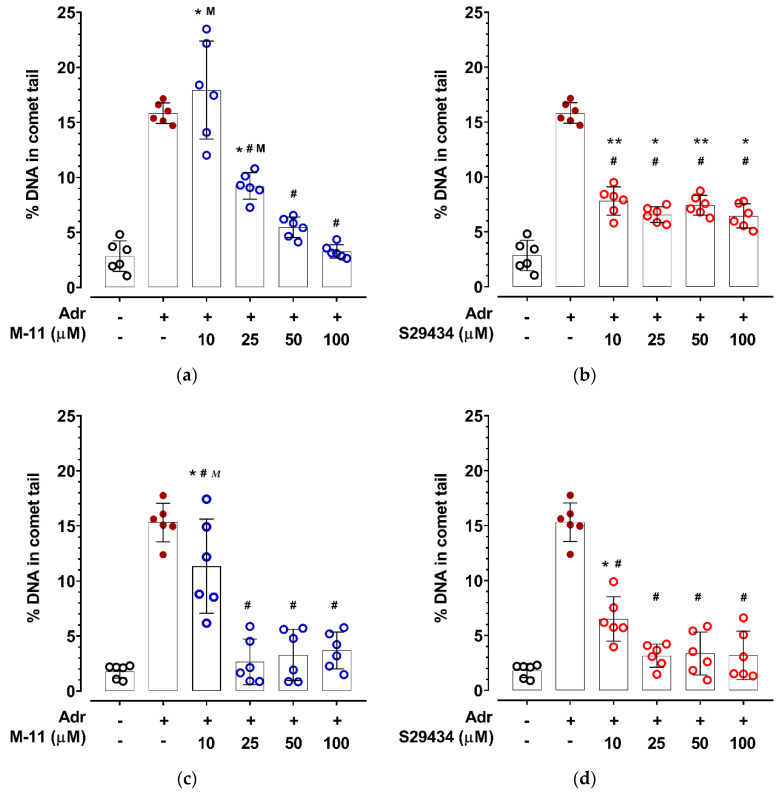
The influence of pre-incubation with NQO2 inhibitors on nuclear DNA damage of HT-22 cells caused by adrenochrome and BNAH. After pre-incubation with inhibitors for 30 or 45 min, cells were exposed to adrenochrome (Adr 125 µM) and BNAH (100 µM) for 30 min. Adrenochrome and BNAH were dissolved in DMSO, final concentration of DMSO was 2%. Data are presented as mean ± S.D. Experimental data were analyzed by one-way ANOVA, Tukey’s multiple comparison test. The percentage of DNA in the comet tail of cells incubated with Adr and BNAH was significantly higher versus the DMSO group (Adr−/inhibitor−) with *p* < 0.01. (**a**) Influence of 30 min pre-incubation with M-11 on nuclear DNA damage (10–100 µM). * *p* < 0.01 vs. DMSO; # *p* < 0.0 vs. Adr+/M-11−; ^M^ *p* < 0.01 vs. other concentrations of M-11; (**b**) influence of 30 min pre-incubation with S29434 on nuclear DNA damage (10–100 µM). * *p* < 0.05, ** *p* < 0.01 vs. DMSO; # *p* < 0.01 vs. Adr+/S29434−; (**c**) influence of 45 min pre-incubation with M-11 on nuclear DNA damage (10–100 µM). * *p* < 0.01 vs. DMSO; # *p* < 0.01 vs. Adr+/M-11−; ^M^ *p* < 0.01 vs. other concentrations of M-11; (**d**) influence of 45 min pre-incubation with S29434 (10–100 µM). * *p* < 0.01 vs. DMSO; # *p* < 0.01 vs. Adr+/S29434−. For details see Table S6.

Considering the ROS production results assessment (Figure 2) and levels of DNA damage caused by co-incubation of cells with the substrate and co-substrate NQO2 (Figure 4), a 125 µM concentration of adrenochrome and a 30 min incubation time were chosen for a quantitative analysis of the effect of NQO2 inhibitors on the nuclear DNA damage of HT-22 cells (Figure 5). Such conditions provide a maximum percentage of DNA in the comet tail, while lacking atypical DNA comets (Figure 4). Incubation of cells with adrenochrome and BNAH for 30 min results in a more than fivefold increase in the average percentage of comet-tail DNA compared to the control groups (Figure S3, Table S4). In the absence of the NQO2 co-substrate BNAH, adrenochrome was unable to affect the comet tail DNA percentage versus vehicle (DMSO), but increased DNA damage versus intact control (intact cells). DMSO or BNAH did not change the percentage of DNA in the comet tail compared to the intact control (Figure S3, Table S4).

Analysis of the concentration dependence of inhibitor action under the selected experimental conditions showed that 30 min pre-incubation of cells with the M-11 compound at 25, 50 and 100 µM concentrations reduces the damage to cell DNA caused by incubation with adrenochrome and BNAH (Figure 5a). Concentrations of 50 and 100 µM of the M-11 compound reduced comet-tail DNA percentage to control values. Under the same experimental conditions, the NQO2 inhibitor S29434 significantly reduced the percentage of comet-tail DNA in the whole range of concentrations (10–100 µM); however, no differences with the control group were observed (Figure 5b). Thus, it was found that after 30 min of HT-22 cells pre-incubation with NQO2 inhibitors, the protective effect of M-11 at 50 and 100 µM concentrations on DNA prevails over the effect of S29434, but is inferior at 10 µM (Figure 5a,b).

An increase in the pre-incubation time with NQO2 inhibitors to 45 min enhances their protective effect on nuclear DNA of HT-22 cells (Figure 5c,d). All used concentrations of M-11 significantly reduced cell DNA damage caused by 30 min incubation with adrenochrome and BNAH (Figure 5c). The compound M-11 at concentrations of 25, 50 and 100 µM reduced the percentage of DNA in the comet tail to control values. Similar to M-11, 10 µM concentration of S29434 reduced the percentage of comet-tail DNA, while 25, 50 and 100 µM concentrations prevented nuclear DNA damage of HT-22 cells caused by incubation with adrenochrome and BNAH (Figure 5d). In control experiments, M-11 and S29434 did not affect DNA damage in HT-22 cells at a maximum concentration of 100 μM in the absence of adrenochrome and BNAH in the incubation medium (Table S5).

**Figure 6:**
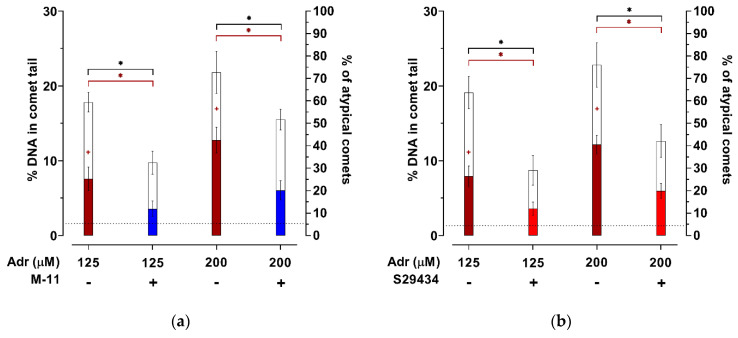
The influence of pre-incubation with NQO2 inhibitors on nuclear DNA damage of HT-22 cells exposed to adrenochrome and BNAH for 120 min. Cells were pre-incubated with M-11 (**a**) or S29434 (**b**) (100 µM) for 30 min, and then were exposed to adrenochrome (125 or 200 µM) and BNAH (100 µM) for 120 min. Adrenochrome and BNAH were dissolved in DMSO. Final concentration of DMSO was 2%. All experiments were performed in 6 replicates. Data are presented as mean ± S.D. The percentage of DNA in the comet tail (blank bars) of cells exposed to adrenochrome and BNAH was significantly higher than DMSO (dotted line) with *p* < 0.001 (one-way ANOVA with Tukey’s post-hoc). Data on the percentage of atypical comets (colored bars) were evaluated using Kruskal-Wallis test with Dunn’s post-hoc: + *p* < 0.01 vs. DMSO. To evaluate the differences between groups exposed to adrenochrome and BNAH, two-way ANOVA with Tukey’s post-hoc was used: * *p* < 0.001 vs. pre-incubation with M-11 or S29434. For details see Tables S7 and S8.

In the further study of the M-11 compound’s effect on DNA damage, a long-term incubation (120 min) of HT-22 cells with adrenochrome and BNAH was performed, where, in addition to typical DNA comets, atypical comets were registered, reflecting a higher degree of DNA fragmentation. The addition of 125 µM or 200 µM of adrenochrome to the incubation media significantly increased the percentage of DNA in the comet tail and the number of atypical DNA comets compared to the control group (DMSO) (Figure 6).

Significant effects of adrenochrome concentration (F = 40.66, *p* < 0.001 for Figure 6a; F = 40.39, *p* < 0.001 for Figure 6b), pre-incubation with M-11 (F = 88.75, *p* < 0.001) or S29434 (F = 81.37, *p* < 0.001) on the percentage of DNA in the comet tail were observed. There was no relationship between these factors (F = 1.33, *p* = 0.26 for Figure 6a; F = 0.89, *p* = 0.42 for Figure 6b). Similar to experiments with 30 or 45 min pre-incubation (Figure 6), 100 µM of M-11 or S29434 were also effective at 120 min incubation with BNAH and adrenochrome at 125 µM, significantly reducing the percentage of DNA in the comet tail (Figure 6). M-11 and S29434 had the same effect on the percentage of comet-tail DNA after 120 min incubation with BNAH and adrenochrome at a 200 µM concentration (Figure 6).

Two-way ANOVA test revealed significant effects of adrenochrome concentration (F = 42.78, *p* < 0.001 for Figure 6a; F = 50.00, *p* < 0.001 for Figure 6b), pre-incubation with M-11 (F = 84.96, *p* < 0.001) or S29434 (F = 127.3, *p* < 0.001) on the percentage of atypical comets. The interaction between adrenochrome concentration and pre-incubation with M-11 was statistically significant (F = 5.38, *p* = 0.031), whereas this interaction for S29434 was not observed (F = 4.03, *p* = 0.058). M-11 and S29434 significantly reduced the percentage of atypical comets under conditions of 120 min incubation of HT-22 cells with BNAH and adrenochrome at both concentrations, leveling out statistically significant differences from the control group (Figure 6).

Thus, the ability of M-11 and S29434 to reduce both the percentage of DNA in the tail of typical comets and the number of atypical comets specific to long-term incubation of HT-22 cells with high concentrations of adrenochrome has been established.

Additionally, an apoptosis analysis was carried out using Annexin V/PI staining to corroborate the protective properties of M-11 in an adrenochrome/BNAH-induced cell damaging model. HT-22 cells were pre-treated with M-11 (100 µM) or S29434 (25 µM) for 45 min and afterwards exposed to adrenochrome (125 µM) and BNAH (100 µM) for 120 min. A significant increase in both early (*p* < 0.001) and late (*p* < 0.001) apoptosis rates was observed following cell treatment with adrenochrome/BNAH (Table 1). M-11 has been shown to decrease the percentage of late apoptotic cells, with no substantial effect on the number of early apoptotic cells, thus indicating that M-11 protects HT-22 cells from adrenochrome/BNAH-initiated apoptosis. Incubation of HT-22 with NQO2 inhibitor S29434 under similar experimental conditions resulted in a more considerable reduction of percentage of late apoptotic cells (*p* < 0.001), while no changes in the number of early apoptotic cells were mentioned. Importantly, M-11, as well as S29434, did not alter the percentage of early and late apoptotic cells when adrenochrome/BNAH were omitted, highlighting compounds’ failure to affect apoptosis in the absence of cell damage.

## 3. Discussion

Data demonstrating NQO2 expression in HT-22 immortalized mouse hippocampal neuronal cells are consistent with those previously reported by Chomarat et al. on immunostaining and enzymatic activity studies, where HT-22 cells were used for cellular knock-down of NQO2 [82]. Expression and enzymatic activity of NQO2 revealed by various approaches, low basal level of NAD(P)H dehydrogenase [quinone] 1 (NQO1; QR1) homologous enzyme [83], ensure the adequacy of using HT-22 cells for modeling oxidative cellular damage in the presence of NQO2 substrate and co-substrate.

NQO2 crystallizes as a homodimer, and the reduction reaction catalysis is of a ping-pong type [36,84]. NQO2 is significantly superior to NQO1 in the reduction of ortho-quinone metabolic products of endogenous catecholamines (aminochrome, adrenochrome, dopachrome) [37,44], which is associated with the specifics of hydrogen bond formation in the active site of the enzyme [36,85]. Unlike NQO1 [86], NQO2 does not recognize NAD(P)H and uses N-alkyl nicotinamide derivatives (NMNH, NRH, BNAH) as hydrogen donors [36]. The given data determine the choice of adrenochrome and BNAH as substrates and co-substrates.

Published data indicate the contribution of NQO2 enzymatic activity to the generation of O_2_^•−^ under conditions of increased generation of enzyme substrates [43,44,87]. In the nervous system, similar processes are distinctive to the NQO2-catalyzed reduction of orthoquinone derivatives of catecholamines, for example, aminochrome [32,42] (Figure 1). Since the rates of cyclization reactions of dopamine o-quinone and its subsequent oxidation significantly exceed the rate of aminochrome rearrangement, the latter can temporarily accumulate in the cytosol [42,88] and, being a substrate of NQO2, contribute to the intensification of oxidative stress [26,27,32,42] (Figure 1). Similar patterns have been demonstrated for norepinephrine [19] and adrenaline [89]. In addition, the contribution of NQO2 to cellular damage may also be associated with the oxidation of the co-substrate NRH to NR+ with subsequent formation of 4-pyridone-3-carboxamide riboside in the presence of O_2_^•−^, which activates cell autophagy in vitro [90]. Therefore, to study ROS production in the presence of adrenochrome and BNAH, the DNA-binding CellROX Green Reagent [91] and CM-H_2_DCFDA [92] dyes were chosen. CellROX Green Reagent is oxidized by O_2_^•−^ and hydroxyl radical (OH^•^), unlike H_2_O_2_, and is used in ROS generation models with menadione (vitamin K3)-exogenous para-quinone with NQO2 substrate properties [93,94]. CM-H_2_DCFDA was used as a general oxidative stress indicator [92].

Our experiments in HT-22 cells showed an increase in CellROX Green Reagent and CM-H_2_DCFDA fluorescence in the presence of the endogenous NQO2 substrate adrenochrome (125 µM) and BNAH (100 µM). The data obtained suggest an increase in O_2_^•−^ production which is consistent with the results of in vitro studies in other cell models. Thus, K562 cell incubation with adrenochrome (100 µM) and BNAH (100 µM) caused an increase in ROS production evaluated through the electron paramagnetic resonance (EPR) spectra intensities [43]. In addition, an increase in ROS production in the presence of the same concentrations of adrenochrome and BNAH was detected on CHO, SHSY-5Y and C3H cell lines, overexpressing NQO2 in comparison with basal expression [43,44].

The ability of the NQO2 inhibitors M-11 (100 µM) and S29434 (25 µM) to prevent an increase in ROS generation caused by incubation of HT-22 cells with adrenochrome and BNAH is consistent with previously demonstrated action of S29434 in vitro [51,81]. It was found in various cell models using the EPR method that pre-incubation with S29434 (20 µM) for 30 min prevented an increase in ROS production caused by adrenochrome (100 µM) and BNAH (100 µM) [43]. Other NQO2 inhibitors (quercetin, resveratrol, imatinib) as well as a specific miRNA silencing of NQO2 had a similar effect on ROS production [49].

In our in vitro study, the ability of endogenous substrate NQO2 adrenochrome to enhance nuclear DNA damage of cells with confirmed expression of NQO2 in the presence of co-substrate BNAH was established for the first time using the comet assay method. An increase in the percentage of DNA in the comet tail and atypical comets upon incubation of HT-22 cells with adrenochrome (50–200 µM) is consistent with the ability of its precursor adrenaline (5–300 µM) to increase the total comet score [95] in human lymphocytes measured by the comet assay after 1 h, 2 h and 4 h of treatment [96]. The authors of the study associate the damaging effects of adrenaline with the formation of oxidative products of adrenaline. This assumption is consistent with the possibility of complete oxidation of adrenaline (10 µM) to adrenochrome within 120 min in a cell-free system and an increase in the formation of 8-oxodG in vitro in the presence of variable charge metals [97]. The contribution of NQO2 to DNA damage of human lymphocytes by quinone derivatives of adrenaline is also confirmed by an increase in the percentage of comet-tail DNA during incubation of cells with menadione under conditions of homologous enzyme NQO1 inhibition [98]. DNA damage of HT-22 cells, caused by the co-incubation of adrenochrome (50–200 µM) and BNAH for up to 120 min, revealed in the current research, is consistent with the ability of adrenochrome to disrupt DNA synthesis and transcription [97,99].

To quantitatively study the effect of NQO2 inhibitors on the DNA damage of HT-22 cells, a concentration of 125 µM of adrenochrome was chosen, under which, when incubated with BNAH, adrenochrome causes ROS production and an increase in the percentage of comet-tail DNA in the absence of atypical comets, which are traditionally analyzed separately [80,95]. In control experiments without BNAH, adrenochrome (125 µM) did not enhance DNA damage, which is consistent with a close to basal level of ROS production upon incubation of K562 cells with adrenochrome at a 100 µM concentration [43].

In our study, the effect of 30 min pre-incubation M-11 at concentrations of 50 and 100 µM was more pronounced compared to S29434 at the same concentrations. At the same time, S29434 was already effective at a concentration of 10 µM. At 45 min pre-incubation the effect of both compounds at concentrations of 25–100 µM was equal. The protective effect of the M-11 compound (25–100 µM) on HT-22 cells DNA when incubated with endogenous substrate of the enzyme adrenochrome and co-substrate BNAH is consistent with our previously published results [73]. Under conditions of NQO1 inhibition, pre-incubation (30 min) of bone marrow cells of ICR mice with the M-11 compound (30 or 50 µM) attenuated menadione-induced DNA damage, more than halving the percentage of DNA in the comet tail [72,73]. It is important to note that the minimum effective concentrations of S29434 compound (10 and 25 μM) identified in this study by the comet assay cause almost complete inhibition of NQO2 in vitro [100]. Moreover, the protective effect of M-11 and S29434 is consistent with their ability to downregulate ROS production in the presence of adrenochrome and BNAH, shown in this work and in the study of Cassagnes et al. [43]. The increase in time of incubation or in concentration of adrenochrome leads to the formation of atypical comets, which reflects severe DNA damage [79,80]. NQO2 inhibitors M-11 and S29434 were able to reduce the number of atypical comets, possibly attenuating this type of ROS-induced DNA damage. Therefore, the action of both compounds on short- and long-term exposure of HT-22 cells to adrenochrome is similar. The lower effectiveness of M-11 compared to S29434 in ROS mitigation, comet assay at 30 min pre-incubation time and apoptosis assay can be explained by a higher affinity of S29434 towards NQO2 and a different lipophilicity of compounds [57,68].

To date, it has been proven that ROS overproduction and oxidative damage to macromolecules, including DNA, are implicated in the pathogenesis of neurodegenerative diseases [101,102]. Therefore, a protective effect of NQO2 inhibitors revealed when incubated with an endogenous substrate adrenochrome resulted in a decrease in both the percentage of DNA in the comet tail and the percentage of atypical comets. This can be considered as a component of the neuroprotective effect under enzyme-inhibition conditions. This assumption is consistent with the data obtained in the apoptosis assay [103,104].

In vitro studies, carried out in the present work, are in agreement with the results of our in vivo studies. In the model of Parkinson’s disease, we have shown neuroprotective effect of M-11 (7.5 mg/kg, i.p.) when the compound was administered over 14 days to ICR mice, starting on the day of 6-OHDA injection [74]. The neuroprotective effect of M-11 is consistent with the ability of the NQO2 inhibitor S29434 to prevent ROS production and cell death induced by paraquat in vitro and in vivo [100,105], as well as to exert a protective effect on dopaminergic neurons when incubated with the active metabolite of 1-methyl-4-phényl-1,2,3,6-tetrahydropyridine MPP+ [81]. The ability of NQO2 inhibitors to cause neuroprotection is in line with the improved learning abilities of *Nqo2*^−/−^ mice in the Morris water maze task [106]. Clinical studies have shown increased expression of the *NQO2* gene in patients with Alzheimer’s [107] and Parkinson’s [108] diseases.

Thus, the present study using HT-22 hippocampal cell culture represents an experimental model for studying the contribution of NQO2 to the neuronal damage in vitro. NQO2 inhibitor M-11, a 2-mercaptobenzimidazole derivative, prevented ROS production and nuclear DNA damage, and decreased the percentage of late apoptotic cells. The protective effect of NQO2 inhibition suggests that this mechanism can be considered as a pharmacological target to ensure neuroprotection, and the M-11 compound is a convenient analytical tool and possible candidate for a new neuroprotective agent development.

## 4. Materials and Methods

### 4.1. Chemicals

M-11 compound (2-[2-(3-oxomorpholin-4-il)-ethylthio]-5-ethoxybenzimidazole hydrochloride) (Figure S4) was synthesized in FSBI “Zakusov Institute of Pharmacology” (Moscow, Russia) as described in [109], adrenochrome (3-hydroxy-1-methyl-2,3-dihydroindole-5,6-dione), bovine serum albumin (BSA), fetal bovine serum (FBS), menadione (2-methylnaphthalene-1,4-dione), dicoumarol (4-hydroxy-3-[(4-hydroxy-2-oxochromen-3-yl)methyl]chromen-2-one), RPMI-1640, glycerin, L-glutamine, sodium deoxycholate, polyacrylamide gel (PAGE), EGTA, EDTA, EDTA-Na_2_, NaCl, NaOH, PBS, PMSF, SDS, Tris-HCl, TBS-T, tert-butyl hydroperoxide (TBHP), propidium iodide (PI), 1X protease inhibitor cocktail (#P8340), 1X phosphatase inhibitor cocktail 2 (#P5726), 1X phosphatase inhibitor cocktail 3 (#P0044) were purchased from Sigma-Aldrich (St-Louis, MO, USA); Triton X-100 and DMSO were purchased from Amresco (Solon, OH, USA); DMEM was purchased from HyClone (Marlborough, MA, USA); S29434 (N-(2-(2-methoxy-6H-dipyrido [2,3-a:3′,2′-e]pyrrolizin-11-yl)ethyl)furan-2-carboxamide) was purchased from MedChemExpress (Monmouth Junction, NJ, USA); BNAH (1-benzyl-1,4-dihydronicotinamide) was purchased from USBiological (Salem, MA, USA); light melting agarose type 4 and high melting agarose type 1 were purchased from Panreac (Barcelona, Spain); SYBR-Green I dye was purchased from Invitrogen (Carlsbad, CA, USA); Pierce BCA Protein Assay Kit (#23225), polyclonal rabbit anti-NQO2 antibodies (1:1000, PA5-96324), CellROX Green Reagent (#C10444), CM-H_2_DCFDA (# C6827) and Annexin V-FITC Apoptosis Detection Kit (#BMS500FI/100) were purchased from Thermo Fisher Scientific (Waltham, MS, USA); SignalFire Elite ECL Reagent, rabbit anti-a-tubulin antibodies (1:1000; #2125), secondary antibody conjugated with HRP (anti-rabbit IgG, 1:12,000, #7074) were purchased from Cell Signaling (Danvers, MA, USA).

### 4.2. HT-22 Immortalized Mouse Hippocampal Neuronal Cell Culture

The HT-22 cell line was a kind gift from Prof. F. Wiegant (Utrecht University, Netherlands) [110]. HT-22 cells were maintained in DMEM medium, 5% FBS, 2 mM *L*-glutamine in 5% CO_2_ and 95% air at 37 °C, and were passaged by trypsinization.

For Western blot analysis and comet assay, HT-22 cells were cultured in 6-well plates (Corning, NY, USA) (1.8 × 105 cells/well) in DMEM containing 5% FBS for 72 h. For flow cytometry detection of ROS production, HT-22 cells were cultured in 12-well plates (Corning, NY, USA) (2 × 105 cells/well) in DMEM, containing 5% FBS for 24 h.

### 4.3. Incubation of HT-22 Cells with Substrate, Co-Substrate and Inhibitors of NQO2

All NQO2 substrates, co-substrate and inhibitors were dissolved in appropriate solvents immediately prior to their addition to the culture medium. Stock solution of NQO2 substrate adrenochrome (200 mM), co-substrate BNAH (100 mM) and inhibitor S29434 (10 mM) were prepared in DMSO, stock solution of another NQO2 inhibitor M-11 (10 mM) was prepared in the growth medium. Serial dilutions of adrenochrome and M-11 were made in the growth medium and S29434 in DMSO. Serial dilutions of BNAH were made in DMSO for comet assay. Adrenochrome was used in final concentrations of 50, 100, 125, 150 and 200 µM. Final concentration of BNAH was 100 µM. HT-22 cells were exposed to adrenochrome and BNAH in combination or separately for 15 min for ROS measurements, 30, 60, 90 and 120 min for comet assay and 120 min for apoptosis assay. Inhibitors M-11 and S29434 at final concentrations of 1.0, 10, 25, 50 and 100 µM were added to culture medium 30 or 45 min prior to the exposure of HT-22 cells to adrenochrome and BNAH. Final concentrations of DMSO were 2% for comet assay, 0.2% for ROS measurement and 0.3% for apoptosis assay. DMSO was used in all experimental groups except for intact cells.

### 4.4. Protein Preparation and Western Blot Analysis

Cells were lysed with RIPA buffer (10 mM Tris-HCl (pH 8.0), 1 mM EDTA, 0.5 mM EGTA, 1% Triton X-100, 0.1% sodium deoxycholate, 0.1% SDS, 0.14 mM NaCl, 1X protease inhibitor cocktail (#P8340), 1 × phosphatase inhibitor cocktail 2 and 3 (#P5726, P0044), 1 mM PMSF). Protein concentration was determined using Pierce BCA Protein Assay Kit. Lysate were loaded on SDS-PAGE and separated proteins were transferred onto nitrocellulose membranes (#GE10600002, Sigma-Aldrich, St-Louis, MO, USA). The membranes were blocked in 5% non-fat milk or 5% BSA in TBS-T for 60 min at room temperature, incubated with the appropriate primary antibody (+4 °C, overnight) and then with a secondary antibody conjugated with HRP (60 min at room temperature). Bands were visualized using SignalFire Plus ECL Reagent. Membranes were scanned using Amersham Imager 680 (GE HealthCare, Chicago, IL, USA) and quantified in Image Quant TL v.8.1 (GE HealthCare, Chicago, IL, USA). Alpha-tubulin was used as loading control.

### 4.5. ROS Measurement

For flow cytometry detection of general ROS production, HT-22 cells were pre-loaded with 5 µM of CM-H_2_DCFDA as a general oxidative stress indicator in serum-free DMEM. After staining procedure (30 min, 37 °C, 5% CO_2_), loading media were replaced by DMEM, containing 5% FBS for CM-H_2_DCFDA cleavage by intracellular esterases (20 min, 37 °C, 5% CO_2_). Cells were exposed to M-11 and S29434 in final concentrations of 10, 25, 50 and 100 µM for 45 min and then to adrenochrome and BNAH for 15 min. As a positive control, 200 µM of TBHP (30 min) was used. The samples were harvested, stained with PI (25 µg/mL; 5 min) and analyzed by NovoCyte 2060 flow cytometer (Acea Biosciences Inc., San Diego, CA, USA), using 488 nm excitation and collecting fluorescence emission; a 530/30 bandpass filter for CM-H_2_DCFDA and a 690/50 BP filter for propidium iodide (PI) dead cell staining. Following application of standard fluorescence compensation technique, medians of fluorescence histogram (MFI) into CM-H_2_DCFDA ^+^/PI^−^ cell populations were used for statistical analysis (15,000 events were collected in each probe gated as “live cells”).

For flow cytometry detection of subcellular ROS production, cells were pre-stained with 5 µM of CellROX Green Reagent according to the manufacturer’s recommendations. After a staining procedure (15 min, 37 °C, 5% CO_2_) media were replaced by DMEM, containing 5% FBS. Cells were exposed to adrenochrome and BNAH for 5 or 15 min. As a positive control, 200 µM of TBHP (30 min) was used. The samples were harvested, stained with PI (25 µg/mL; 5 min) and analyzed by NovoCyte 2060 flow cytometer (Acea Biosciences Inc., San Diego, CA, USA), using 488 nm excitation and collecting fluorescence emission in median fluorescence intensity (MFI); a 530/30 bandpass filter for CellROX Green Reagent and a 690/50 BP filter for PI dead cell staining. Following application of standard fluorescence compensation technique, medians of fluorescence histogram (MFI) into CellROX Green ^+^/PI^−^ cell populations were used for statistical analysis (15,000 events were collected in each probes gated as “live cells”).

### 4.6. Apoptosis Assay

For the detection of apoptotic stages, HT-22 cells were treated with M-11 (100 µM), S29434 (25 µM) for 45 min and then with adrenochrome and BNAH for an additional 120 min. After the treatment cells were washed with PBS and incubated in DMEM, containing 5% FBS for 24 h, cells were harvested and stained with Annexin V-FITC Apoptosis Detection Kit according to the manufacturer’s recommendations. The samples were analyzed by NovoCyte 2060 flow cytometer (Acea Biosciences Inc., San Diego, CA, USA), using 488 nm excitation and collecting fluorescence emission: a 530/30 bandpass filter for Annexin V-FITC and a 690/50 BP filter for PI dead cell staining. Following application of standard fluorescence compensation technique, cell percentages of Annexin V/PI dual parameter dot plot were used for statistical analysis (10,000 events were collected in each probe).

### 4.7. Single-Cell Gel Electrophoresis Assay (Alkaline Comet Assay)

All the procedures were conducted under dim light to prevent the occurrence of additional DNA damage. After the incubation with adrenochrome and after BNAH cells were washed in 1 mL of PBS, 500 µL of PBS was added into the well and cells were scraped off the plate, transferred into an Eppendorf tube and suspended. After that, 35 µL of cell suspension was intensely mixed with 175 µL of 0.9% light melting agarose solution. The same amount of obtained mixture was dropped on slides pre-coated with 1% of high melting agarose. The slides were covered with coverslips and placed on ice for 5 min. After gel solidification coverslips were gently removed and slides were placed into a Schifferdecker-type glass cuvette filled with ice cold lysis solution (10 mM Tris-HCl, 2,5 M NaCl, 100 mM EDTA-Na_2_, 1% Triton X-100, 10% DMSO, pH 10, 4 °C) and incubated at 4 °C for at least 1 h. After the lysis step, the slides were washed in deionized water and placed into a cuvette filled with alkaline buffer (300 mM NaOH, 1 mM EDTA-Na_2_, pH > 13, 4 °C) for 20 min. After the denaturation step, slides were transferred to the electrophoresis chamber (BioRad, Hercules, CA, USA), filled with 2.2 L of the same solution. Electrophoresis was performed for 20 min at electric field strength of 1.0 V/cm; the applied voltage was 32 V and the current was 400 mA. After electrophoresis, the slides were washed in 1 × PBS, fixed in 70% ethanol at room temperature. Then, drying slides were stained with SYBR Green I (1:10,000 in TE buffer/glycerin (1:1 *v/v*), pH 8.5) for 30 min in the dark. Analysis was performed on a Mikmed-2 12T epifluorescence microscope (“LOMO”, St. Petersburg, Russia) combined with a high-resolution digital camera (VEC-335, St. Petersburg, Russia), at 100× magnification. The images of comets were analyzed using CASP v.1.2.3 software (www.casplab.com). The DNA damage was evaluated by the percentage of DNA in the tail of the comet (% tDNA). Spontaneous DNA damage was measured in intact cells incubated in cultural medium only. Control groups included cells incubated with 2% DMSO, adrenochrome or BNAH. Each experimental group was characterized by mean and standard deviation of medians of % tDNA obtained as a result of analysis of at least 100 non-atypical comets per slide. The percentage of atypical comets was counted as ratio of non-overlapping comets with barely visible or invisible nucleoid head and large, broad tail to the total amount of non-overlapping comets. For examples of comets used in the analysis, see Figure S2. All experiments were performed in six repetitions.

### 4.8. Experimental Animals

The study was performed in male ICR (CD-1) mice (25–30 g, n = 5) obtained from Pushchino Breeding Center (Branch of the Institute of Bioorganic Chemistry, Russian Academy of Sciences). Animals were housed under standard vivarium conditions (20–22 °C, 30–70% humidity, 12 h light/dark cycle) in plastic cages with sawdust bedding and 5 animals per cage.

### 4.9. Ethical Approval

All experimental procedures were approved by the bioethics committee of the FSBI “Zakusov Institute of Pharmacology”, protocol #08 of 30 September 2021. All applicable national [111] and international [112] guidelines for care and use of experimental animals were followed.

### 4.10. ICR Mice Bone Marrow Cells

Mice were sacrificed by cervical dislocation. Epiphysis of femurs were cut off and bone marrow cells were flushed with 3 mL of RPMI-1640 medium containing 10% FBS per bone. The sample from each animal was divided in 300 µL aliquots and pre-incubated at 37 °C in the presence or absence of 10 µM dicoumarol with DMSO or M-11 in final concentrations of 5, 10, 15, 30 and 50 µM for 30 min. After pre-incubation suspension of bone marrow cells was incubated with menadione at a final concentration of 25 µM for 60 min at 37 °C. After exposition to menadione alkaline comet assay was conducted as described in Section 4.6 with slight modifications, i.e., after the washing procedure, bone marrow cells were resuspended in 300 µL of PBS. 

Stock solutions of M-11, dicoumarol and menadione were prepared immediately prior to their addition to incubation medium. Stock solution of M-11 was prepared and diluted in RPMI. Solutions of dicoumarol and menadione were prepared in DMSO. Final concentration of DMSO did not exceed 2%. All experiments were performed in five repetitions.

### 4.11. Statistical Analysis

To evaluate the experimental data distribution, D’Agostino-Pearson and Shapiro-Wilk tests were used. Statistical significance was calculated using one- or two-way ANOVA with Tukey’s post-hoc test or Kruskal-Wallis test with Dunn’s post-hoc test. Data are presented as the mean and standard deviation (mean ± S.D.) or median with minimum and maximum (Mdn (min–max)). A value of *p* < 0.05 was considered to be statistically significant. Statistical analysis and visualization were performed using GraphPad Prism software version 8.0.1 for Windows (GraphPad, La Jolla, CA, USA, www.graphpad.com).

## Figures and Tables

**Table 1:** The impact of M-11 and S29434 on apoptosis of HT-22 cells treated with adrenochrome and BNAH.

	Apoptotic Stages (% of Cells)	
	Early	Late
0.3% DMSO	2.7 ± 0.3	0.2 ± 0.1
Adrenochrome/BNAH	30.9 ± 2.5 ***	41.4 ± 0.01 ***
M-11 (100 μM) + Adrenochrome/BNAH	32.3 ± 4.8	35.7 ± 0.5 ^###^
S29434 (25 μM) + Adrenochrome/BNAH	32.5 ± 2.2	19.4 ± 0.9 ^###^
M-11 (100 μM)	2.3 ± 0.8	0.5 ± 0.07
S29434 (25 μM)	2.8 ± 0.09	0.6 ± 0.03

HT-22 cells were treated with M-11 (100 µM) or with S29434 (25 µM) for 45 min followed by incubation with adrenochrome and BNAH for an additional 120 min, after which cells were washed with PBS and incubated in DMEM, containing 5% FBS for 24 h. All experiments were performed in 9 replicates. Data shown as mean ± SD (*** *p* < 0.001 vs. 0.3% DMSO; ### *p* < 0.001 compared with “Adrenochrome/BNAH” group; one-way ANOVA with Tukey’s post-hoc).

## Data Availability

All data are presented within the manuscript and Supplementary Materials or are available on request from the corresponding authors.

## Supplementary Materials

The following are available online at https://www.mdpi.com/article/10.3390/ijms222313061/s1.

## Abbreviations

6-OHDA	6-Hydroxydopamine: 5-(2-aminoethyl)benzene-1:2:4-triol
Afobazole	5-Ethoxy-2-[2-(morpholino)-ethylthio]benzimidazole dihydrochloride
BNAH	1-Benzyl-1,4-dihydronicotinamide
BSA	Bovine serum albumin
C3H cells	Murine embryo fibroblast cells
CHO cells	Chinese hamster ovary cells
CM-H_2_DCFDA	acetic 2-[3,6-bis(acetyloxy)-2,7-dichloro-9H-xanthen-9-yl]-4-(chloromethyl)benzoic anhydride
DMAT	4,5,6,7-Tetrabromo-N,N-dimethyl-1H-benzimidazol-2-amine
DMEM	Dulbecco’s Modified Eagle Medium
DMSO	Dimethyl sulfoxide
EDTA	Ethylenediaminetetraacetic acid
EDTA-Na_2_	Ethylenediaminetetraacetic acid disodium salt dihydrate
EGTA	Ethylenebis(oxyethylenenitrilo)tetraacetic acid
EPR	Electron paramagnetic resonance
FBS	Fetal bovine serum
GAPDH	Glyceraldehyde-3-phosphate dehydrogenase
HRP	Horseradish peroxidase
HT-22	Immortalized mouse hippocampal cells
ICR	Institute of Cancer Research outbred mice
K562 cells	Immortalized human myeloid leukemia cells
M-11	2-[2-(3-Oxomorpholin-4-il)-ethylthio]-5-ethoxy benz-imidazole hydrochloride
MAO-A	Amine oxidase [flavin-containing] A
MPP+	N-Methyl-4-phenylpyridine
MT_1_ receptor	Melatonin receptor type 1
MT_3_ receptor	Melatonin dependent regulatory site of NQO2; melatonin receptor type 3
NADH	Dihydronicotinamide-adenine dinucleotide
NADPH	Dihydronicotinamide-adenine dinucleotide phosphate
NMNH	1-(5-O-phosphono-beta-D-ribofuranosyl)-1,4-dihydropyridine-3-carboxamide
NQO1	NAD(P)H dehydrogenase [quinone] 1; QR1
NQO2	Ribosyldihydronicotinamide dehydrogenase [quinone]; NRH: quinone reductase 2; QR2
NR+	N-Ribosylnicotinamide
NRH	1-(beta-D-Ribofuranosyl)-1,4-dihydronicotinamide
O_2_^•^^−^	Superoxide anion radical
OH^•^	Hydroxyl radical
PAGE	Polyacrylamide gel
PBS	Phosphate buffered saline
PI	Propidium iodide
PMSF	Phenylmethylsulfonyl fluoride
ROS	Reactive oxygen species
RPMI-1640	Roswell Park Memorial Institute medium
S29434	N-(2-(2-Methoxy-6H-dipyrido[2,3-a:3′,2′-e]pyrrolizin-11-yl)ethyl)furan-2-carboxamide; NMDPEF
SDS	Sodium dodecyl sulfate
SH-SY5Y cells	Human bone marrow biopsy-derived neuroblastoma cells
Sigma1R	Sigma non-opioid intracellular receptor 1, chaperone Sigma1R
TBB	4,5,6,7-Tetrabromobenzotriazole
TBBz	4,5,6,7-Tetrabromobenzimidazole
TBHP	tert-Butyl hydroperoxide
TBS-T	Tris buffered saline-Tween 20
VAT2	Synaptic vesicular amine transporter; VMAT2
V-ATPase	V-type proton ATPase

## Appendix A

To study the influence of M-11 on NQO1, we utilized a pharmacological approach based on the comparison of M-11 and dicoumarol effects on oxidative damage of nuclear DNA caused by NQO1 and NQO2 substrate menadione. In the used concentration of 10 µM dicoumarol totally inhibits NQO1, while reducing NQO2 activity for only 20% [52]. On bone marrow cells of ICR mice, we have reproduced an increase of DNA damage in the presence of dicoumarol (F = 31.57, *p* < 0.001; Figure A1) [72], which is consistent with Woods et al. [98] and provides evidence of the protective role of NQO1 [113]. We established a link between increased nuclear DNA damage and NQO2 activity earlier, using M-11 [72]. Figure A1 indicates that NQO2 inhibitor M-11 in 5–50 µM range does not cause the enhancement of DNA damage characteristic for NQO1 inhibition in this experimental model. The protective effect of M-11 (F = 46.83, *p* < 0.001) in concentrations of 30 and 50 µM manifested only under conditions of NQO1 inhibition by dicoumarol (interaction: F = 8.28, *p* < 0.001), which is consistent with the protective effect of M-11, starting from a 25 µM concentration, described in the current study. These data suggest the absence of any impact of M-11 in the used concentration range on NQO1 activity.
