# Supplementary material for: Neuroprotective Properties of Quinone Reductase 2 Inhibitor M-11, a 2-Mercaptobenzimidazole Derivative
Source: Int J Mol Sci. 2021 Dec 2;22(23):13061. doi: 10.3390/ijms222313061 (PMC8658107; doi:10.3390/ijms222313061)
Supplement: Supplementary file 1 [file ijms-22-13061-s001.zip › ijms-1494373-supplementary.pdf]

Supplementary Materials

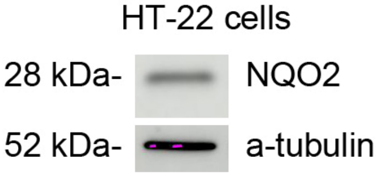

Supplementary Figure S1. NQO2 protein expression in HT-22 cells. HT-22 cells were incubated in 5% FBS. Cells were collected, protein extracts were subjected to polyacrylamide gel electrophoresis and transferred for western blotting. Blots were probed with anti-NQO2 and anti-a-tubulin antibody. The figure shows data from one independent experiment (n=3).

Supplementary Table S1. The effect of adrenochrome and BNAH on ROS production in HT-22 cells.

	Concentration of adrenochrome (µM)	CellROX Green fluorescence (MFI) 5 min	CellROX Green fluorescence (MFI) 15 min	CM-H_2DCFDA (MFI) 15 min
**DMSO (without BNAH)**		4132 (3832 – 4574)	5186 (4771 – 5663)	75695 (74587 – 76874)
**Adrenochrome (µM) + BNAH**	**0**	3793 (3728 – 3842)	5369 (5268 – 5492)	77582 (75984 – 78681)
	**50**	5448 (5029 – 6064)	6415 (6212 – 6694)	126562 (124684 – 129875)
	**100**	6053 (5717 – 6644)	7154 (6637 – 7803) # p = 0.016	132057 (126485 – 135311)
	**125**	8790 (8453 – 8843) * p = 0.003	7530 (7486 – 7886) # p = 0.002	167939 (165987 – 169457) # p=0.03
	**150**	5049 (4766 – 5472)	6956 (6616 – 7307)	169895 (168479 – 171665) # p=0.0003
	**200**	4811 (4611 – 5140)	5924 (5649 – 6299)	169958 (168482 – 172695) # p=0.0001
**TBHP (200 µM)**		21056 (20992-21929)		494311 (492453-499749)

Cells were incubated with adrenochrome (50 – 200 µM) and/or BNAH (100 µM) for 5 and 15 min. Final concentration of DMSO was 0.2 %. TBHP (200 µM; 30 min) was used as positive control. Data are presented as median (min – max). All experiments were performed in 12 replicates. Kruskal-Wallis test with Dunn's post-hoc: *p – statistical significance vs. **Adrenochrome (0µM) + BNAH** group in ROS measurement with CellROX Green Reagent; #p – statistical significance vs. **Adrenochrome (0µM) + BNAH** group in ROS measurement with CM-H_2DCFDA.

Supplementary Table S2. The effect of NQO2 inhibitors on the level of ROS elicited by adrenochrome and BNAH in HT-22 cells.

	Concentration of NQO2 inhibitors (µM)	CellROX Green fluorescence (MFI)	CM-H_2DCFDA (MFI)

**DMSO (without BNAH)**		2210 (2145 – 2306)	51522 (50468 – 52112)
**Adrenochrome + BNAH**		3281 (3160 – 3360) * p=0.003	77657 (76485 – 78535) * p=0.001
**M11/Adrenochrome + BNAH**	**10**	3147 (3106 – 3185)	79046 (78462 – 79344)
	**25**	3161 (3155 – 3166)	72147 (71984 – 72256)
	**50**	2943 (2848 – 3005)	68426 (67943 – 68945)
	**100**	1824 (1797 – 1848) # p<0.001	57206 (56750 – 57882) + p=0.04
**S29434/Adrenochrome + BNAH**	**1**	2625 (2528 – 2688)	60572 (59658 – 61257)
	**10**	2297 (2212 – 2352)	48147 (47421 – 48692) + p=0.046
	**25**	1805 (1738 – 1848) # p=0.0012	40381 (39772 – 40838) + p=0.0012
	**50**	1286 (1239 – 1317) # p<0.001	37275 (36713 – 37697) + p<0.001
**TBHP (200 μM)**		21926 (21113-22535)	469775 (398759-498576)

Cells were pre-incubated with M-11 (10-25-50-100 μM) or S29434 (1-10-25-50 μM) for 45 min. Upon pre-incubation cells were incubated with a combination of adrenochrome (125 μM) and BNAH (100 μM) for 15 min. %. TBHP (200 μM; 30 min) was used as positive control. All experiments were performed in 9 replicates. Data are presented as median (min - max). Kruskal-Wallis test with Dunn's post-hoc: *p – statistical significance vs. corresponding **DMSO** groups; #p – statistical significance vs. **Adrenochrome + BNAH** group in ROS measurement with CellROX Green Reagent; +p – statistical significance vs. **Adrenochrome + BNAH** group in ROS measurement with CM-H_2DCFDA.

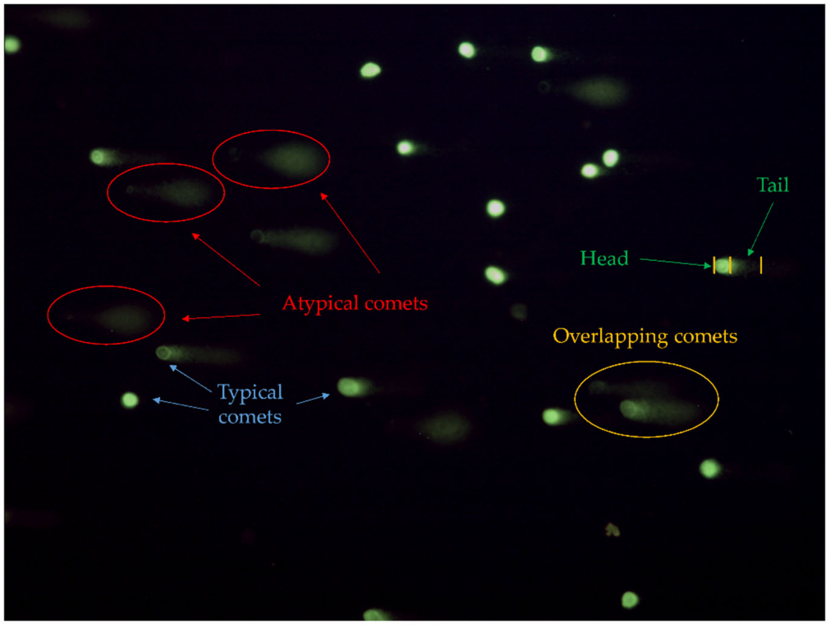

Supplementary Figure S2. Classification of cells on the comet image. **Typical comets** – an example of cells with clearly visible head and tail that were used for calculation of DNA percentage in the comet tail. **Atypical comets** – an example of cells with barely visible or invisible nucleoid head and large, broad tail, which were used for atypical comets percentage count. **Overlapping comets** – comets not included in either type of analysis.

Supplementary Table S3. Dependencies of DNA damage on exposition time and concentration of adrenochrome and BNAH in HT-22 cells.

	**Incubation time, min**	**% of DNA in the comet tail**	**% of atypical comets**
**Intact control (without BNAH)**		1.29 ± 0.42	0.0
**DMSO (without BNAH)**	**120**	1.74 ± 1.11	0.0
**Adrenochrome 50 µM**	**30**	2.59 ± 1.29	0.0
	**60**	2.96 ± 1.79	0.0
	**90**	2.36 ± 0.67	0.0
	**120**	6.39 ± 0.61	2.13 ± 1.59
**Adrenochrome 100 µM**	**30**	9.35 ± 1.97<br>* $p < 0.001$<br>$p^{50} = 0.008$	0.0
	**60**	9.33 ± 2.66<br>* $p < 0.001$<br>$p^{50} = 0.012$	0.0
	**90**	9.6 ± 3.57<br>* $p < 0.001$<br>$p^{50} = 0.004$	0.0
	**120**	12.36 ± 2.77<br>* $p < 0.001$<br>$p^{50} = 0.0026$	13.57 ± 3.0
**Adrenochrome 150 µM**	**30**	21.16 ± 1.81<br>* $p < 0.001$<br>$p^{50} < 0.001$<br>$p^{100} < 0.001$	11.53 ± 1.93
	**60**	20.76 ± 3.97<br>* $p < 0.001$<br>$p^{50} < 0.001$<br>$p^{100} < 0.001$	22.78 ± 8.74<br>+ $p = 0.016$<br>$^{30}p = 0.037$

	**90**	20.17 ± 1.1<br>* $p < 0.001$<br>$p^{50} < 0.001$<br>$p^{100} < 0.001$	21.78 ± 2.67<br>+ $p = 0.02$
	**120**	22.62 ± 2.6<br>* $p < 0.001$<br>$p^{50} < 0.001$<br>$p^{100} < 0.001$<br>$^{30}p = 0.02$<br>$^{90}p = 0.049$	28.2 ± 4.25<br>+ $p = 0.002$<br>$^{30}p < 0.001$
**Adrenochrome 200 μM**	**30**	22.29 ± 2.51<br>* $p < 0.001$<br>$p^{50} < 0.001$<br>$p^{100} < 0.001$	14.5 ± 2.35
	**60**	23.83 ± 4.17<br>* $p < 0.001$<br>$p^{50} < 0.001$<br>$p^{100} < 0.001$	30.85 ± 3.75<br>+ $p < 0.001$<br>$^{30}p < 0.001$
	**90**	25.51 ± 3.77<br>* $p < 0.001$<br>$p^{50} < 0.001$<br>$p^{100} < 0.001$<br>$p^{150} < 0.001$	30.0 ± 7.32<br>+ $p = 0.001$<br>$^{30}p = 0.001$
	**120**	23.11 ± 3.75<br>* $p < 0.001$<br>$p^{50} < 0.001$<br>$p^{100} < 0.001$	36.48 ± 9.88<br>+ $p < 0.001$<br>$^{30}p < 0.001$

Cells were incubated with adrenochrome (50 – 200 μM) and BNAH (100 μM) for 30, 60, 90 and 120 min. Adrenochrome and BNAH were dissolved in DMSO. Final concentration of DMSO was 2%. All experiments were performed in 6 replicates. Data are presented as mean ± S.D. Data on the percentage of DNA in the comet tail: One-way ANOVA with Tukey's post-hoc: * p – vs. **DMSO**. Two-way ANOVA with Tukey's post-hoc: p^{50} – vs. A**drenochrome 50 μM** at the same time point; p^{100} – vs. **Adrenochrome 100 μM** at the same time point; p^{150} – vs. A**drenochrome 150 μM** at the same time point. Data on the percentage of atypical comets: Kruskal-Wallis test with Dunn's post-hoc: + p – vs. **DMSO**. Two-way ANOVA with Tukey's post-hoc (groups with the presence of atypical comets at all time points were taken for comparison, i.e. **Adrenochrome 150** and **200 μM)**: ^{30}p – vs. 30 min incubation with the same concentration of adrenochrome.

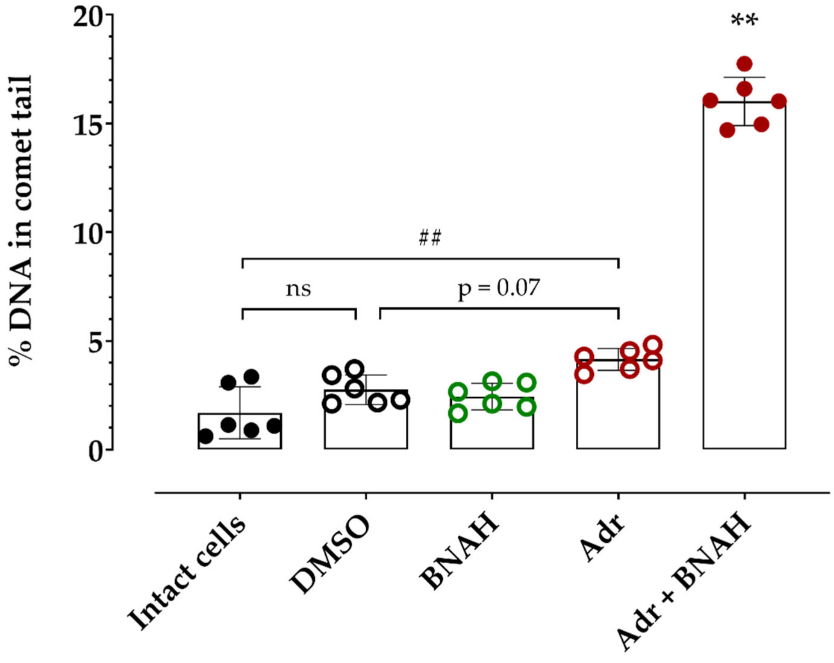

Supplementary Figure S3. The effect of 30 min incubation of hippocampal HT-22 cells with adrenochrome and BNAH on DNA damage. Cells were incubated with adrenochrome (125 µM), BNAH (100 µM) or its combination for 30 min. Adrenochrome and BNAH were dissolved in DMSO, final concentration of DMSO was 2%. All experiments were performed in 6 replicates. Data are presented as mean ± S.D. One-way ANOVA, Tukey multiple comparison test statistical significance: ** p < 0.001 vs. **Intact cells**, **DMSO**, **BNAH** or **Adr** groups. # p < 0.05, ## p < 0.01 vs. **Intact cells** or **BNAH** groups.

Supplementary Table S4. The effect of 30 min incubation of HT-22 cells with adrenochrome and BNAH on DNA damage.

	**% of DNA in the comet tail**
**Intact control**	1.69 ± 1.2
(without BNAH)	* p < 0.001
	# p < 0.001
**DMSO**	2.76 ± 0.68
(without BNAH)	* p < 0.001
**BNAH 100 µM**	2.44 ± 0.61
	* p < 0.001
	# p = 0.017
**Adrenochrome 125 µM**	4.15 ± 0.5
(without BNAH)	* p < 0.001
**Adrenochrome 125 µM + BNAH 100 µM**	16.02 ± 1.11

Cells were incubated with adrenochrome (125 µM), BNAH (100 µM) or their combination for 30 min. Adrenochrome and BNAH were dissolved in DMSO. Final concentration of DMSO was 2%. All experiments were performed in 6 replicates. Data are presented as mean ± S.D. One-way ANOVA with Tukey's post-hoc: * p – vs. **DMSO**, **Adrenochrome 125 µM** or **BNAH 100 µM**. # p – vs. **Adrenochrome 125 µM**.

Supplementary Table S5. The effect of 45 min pre-incubation of intact HT-22 cells with NQO2 inhibitors on DNA damage.

	**% of DNA in comet tail**
**Intact control**	1.7 ± 0.84
**DMSO**	1.97 ± 0.83
**M-11 100 μM**	2.55 ± 0.92
**S29434 100 μM**	2.3 ± 0.88

Cells were incubated with M-11 (100 μM) or S29434 (100 μM) for 45 min in presence of 1% DMSO, after addition of another 1% DMSO cells were incubated for 30 min. All experiments were performed in 6 replicates. Data are presented as mean ± S.D. One-way ANOVA with Tukey's post-hoc: there was no significant difference in the percentage of DNA in the comet tail between groups of used inhibitors and **Intact control** or **DMSO** groups.

Supplementary Table S6. The influence of pre-incubation with NQO2 inhibitors on nuclear DNA damage of HT-22 cells caused by adrenochrome and BNAH.

	**Concentration of NQO2 inhibitors (μM)**	**% of DNA in the comet tail** 30 min	45 min
**Intact control**		2.15 ± 0.99	1.18 ± 1.14
**DMSO**		2.84 ± 1.38	1.79 ± 0.63
**BNAH**		2.24 ± 0.86	2.48 ± 0.78
**Adrenochrome + BNAH**		15.83 ± 0.93<br>* $p < 0.001$	15.31 ± 1.75<br>* $p < 0.001$
**M-11/Adrenochrome + BNAH**	**10**	17.93 ± 4.45<br>* $p < 0.001$<br>^{S} $p < 0.001$<br>^{M} $p < 0.001$	11.34 ± 4.28<br>* $p < 0.001$<br>^{S} $p = 0.0095$<br>^{M} $p < 0.001$
	**25**	9.23 ± 1.2<br>* $p < 0.001$<br># $p < 0.001$<br>^{M} $p < 0.01$	2.66 ± 2.07<br># $p < 0.001$
	**50**	5.46 ± 0.93<br># $p < 0.001$	3.28 ± 2.33<br># $p < 0.001$
	**100**	3.27 ± 0.62<br># $p < 0.001$<br>^{S} $p = 0.047$	3.69 ± 1.67<br># $p < 0.001$
**S29434/Adrenochrome + BNAH**	**10**	7.81 ± 1.28<br>* $p < 0.001$<br># $p < 0.001$	6.51 ± 2.02<br>* $p = 0.013$<br># $p < 0.001$
	**25**	6.57 ± 0.72<br>* $p = 0.009$<br># $p < 0.001$	3.16 ± 1.06<br># $p < 0.001$
	**50**	7.43 ± 0.9<br>* $p < 0.001$<br># $p < 0.001$	3.36 ± 1.96<br># $p < 0.001$
	**100**	6.45 ± 1.11<br>* $p = 0.013$<br># $p < 0.001$	3.18 ± 2.21<br># $p < 0.001$

After pre-incubation with inhibitors for 30 or 45 min, cells were exposed to adrenochrome (125 μM) and BNAH (100 μM) for 30 min. Adrenochrome and BNAH were dissolved in DMSO. Final concentration of DMSO was 2%. All experiments were performed in 6 replicates. Data are presented as mean ± S.D. One-way ANOVA with Tukey's post-hoc: * - vs. **DMSO**; # - vs. **Adrenochrome + BNAH**; ^{M} – vs. other concentrations of **M-11**; ^{S} – vs. equal concentration of **S29434**.

Supplementary Table S7. The influence of pre-incubation with M-11 on nuclear DNA damage of HT-22 cells exposed to adrenochrome with BNAH for 120 min.

	**% of DNA in comet tail**	**% of atypical comets**
**Intact control** (without BNAH)	0.75 ± 0.41	0
**DMSO** (without BNAH)	1.61 ± 1.35	0
**Adrenochrome 125 μM + BNAH**	17.82 ± 1.31<br>* $p < 0.001$<br>$p^{200} = 0.007$	25.33 ± 5.27<br>+ $p = 0.008$<br>$p^{200} < 0.001$
**M-11/Adrenochrome 125 μM + BNAH**	9.73 ± 1.55<br>* $p < 0.001$<br>$p^{125} < 0.001$<br>$^{M}p < 0.001$	11.9 ± 3.43<br>$p^{125} < 0.001$<br>$^{M}p = 0.034$
**Adrenochrome 200 μM + BNAH**	21.82 ± 2.83<br>* $p < 0.001$	42.57 ± 5.72<br>+ $p < 0.001$
**M-11/Adrenochrome 200 μM + BNAH**	15.49 ± 1.38<br>*$p < 0.001$<br>$p^{200} < 0.001$	20.12 ± 4.32<br>$p^{200} < 0.001$

Cells were pre-incubated with M-11 (100 μM) for 30 min, and then exposed to adrenochrome (125 or 200 μM) and BNAH (100 μM) for 120 min. Adrenochrome and BNAH were dissolved in DMSO. Final concentration of DMSO was 2%. All experiments were performed in 6 replicates. Data are presented as mean ± S.D. Data on the percentage of DNA in the comet tail: One-way ANOVA with Tukey's post-hoc: * – vs. **DMSO**. Data on the percentage of atypical comets: Kruskal-Wallis test with Dunn's post-hoc: + – vs. **DMSO**. Two-way ANOVA with Tukey's post-hoc: Statistical significance: ^{125}p – vs. **Adrenochrome 125 μM + BNAH**; ^{200}p – vs. **Adrenochrome 200 μM + BNAH;** ^{M} – vs. **M-11/Adrenochrome 200 μM + BNAH**.

Supplementary Table S8. The influence of pre-incubation with S29434 on nuclear DNA damage of HT-22 cells exposed to adrenochrome with BNAH for 120 min.

	**% of DNA in comet tail**	**% of atypical comets**
**Intact control** (without BNAH)	0.75 ± 0.41	0
**DMSO** (without BNAH)	1.61 ± 1.35	0
**Adrenochrome 125 μM + BNAH**	19.13 ± 2.16<br>* $p < 0.001$<br>$p^{200} = 0.011$	26.42 ± 4.59<br>+ $p = 0.001$<br>$p^{200} < 0.001$
**S29434/Adrenochrome 125 μM + BNAH**	8.71 ± 1.97<br>* $p < 0.001$<br>$p^{125} < 0.001$<br>$^{S}p = 0.024$	11.97 ± 2.99<br>$p^{125} < 0.001$<br>$^{S}p = 0.019$
**Adrenochrome 200 μM + BNAH**	23.46 ± 2.06<br>* $p < 0.001$	40.56 ± 4.13<br>+ $p < 0.001$
**S29434/Adrenochrome 200 μM + BNAH**	12.64 ± 2.19<br>*$p < 0.001$<br>$p^{200} < 0.001$	19.85 ± 3.35<br>$p^{200} < 0.001$

Cells were pre-incubated with S29434 (100 μM) for 30 min, and then exposed to adrenochrome (125 or 200 μM) and BNAH (100 μM) for 120 min. Adrenochrome and BNAH were dissolved in DMSO. Final concentration of DMSO was 2%. All experiments were performed in 6 replicates. Data are presented as mean ± S.D. Data on the percentage of DNA in the comet tail: One-way ANOVA with Tukey's post-hoc: * – vs. **DMSO**. Data on the percentage of atypical comets: Kruskal-Wallis test with Dunn's post-hoc: + – vs. **DMSO**. Two-way ANOVA with Tukey's post-hoc: Statistical significance: ^{125}p – vs. **Adrenochrome 125 μM + BNAH**; ^{200}p – vs. **Adrenochrome 200 μM + BNAH;** ^{S} – vs. **S29434/Adrenochrome 200 μM + BNAH**.

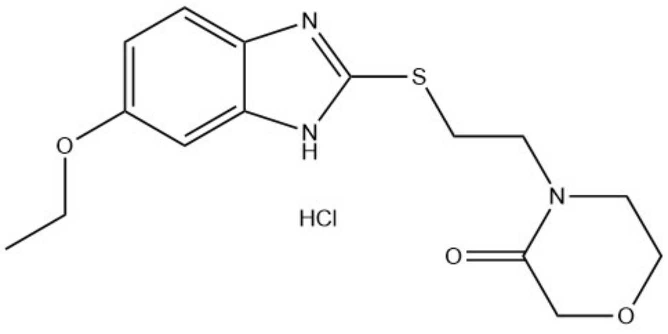
N
S
NH
O
HCl
N
O
O

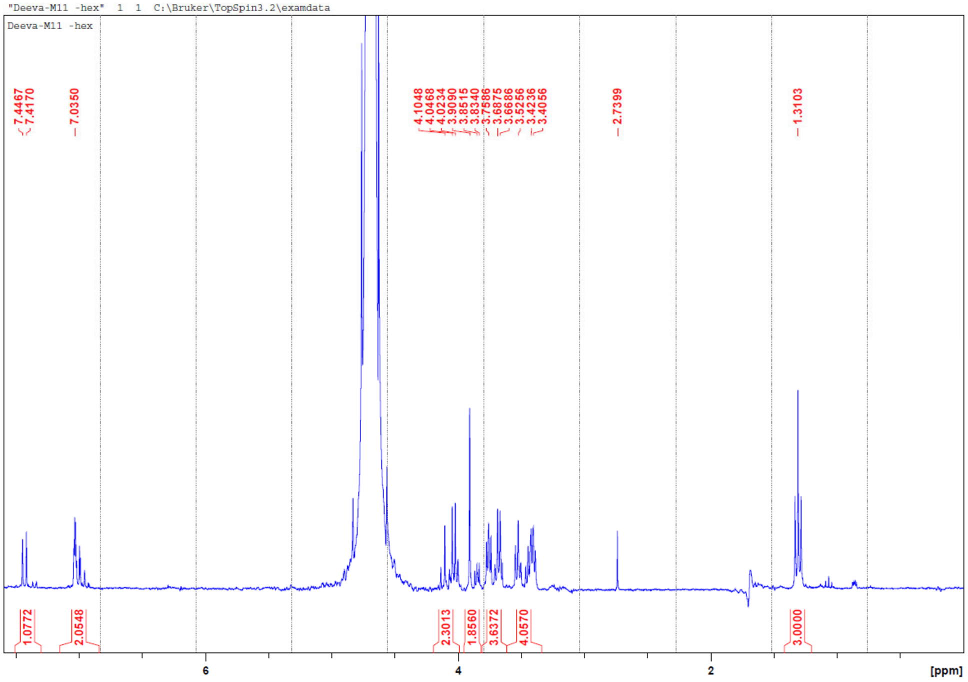
"Deeva-M11 -hex" 1 1 C:\Bruker\TopSpin3.2\examdata
Deeva-M11 -hex
7.4467
7.4170
7.0350
4.1048
4.0468
4.0234
3.9090
3.8515
3.8340
3.7586
3.6875
3.6676
3.5256
3.4236
3.4056
2.7399
1.3103
1.0772
2.0548
2.3013
1.8560
3.6372
4.0570
3.0000
6
4
2
[ppm]

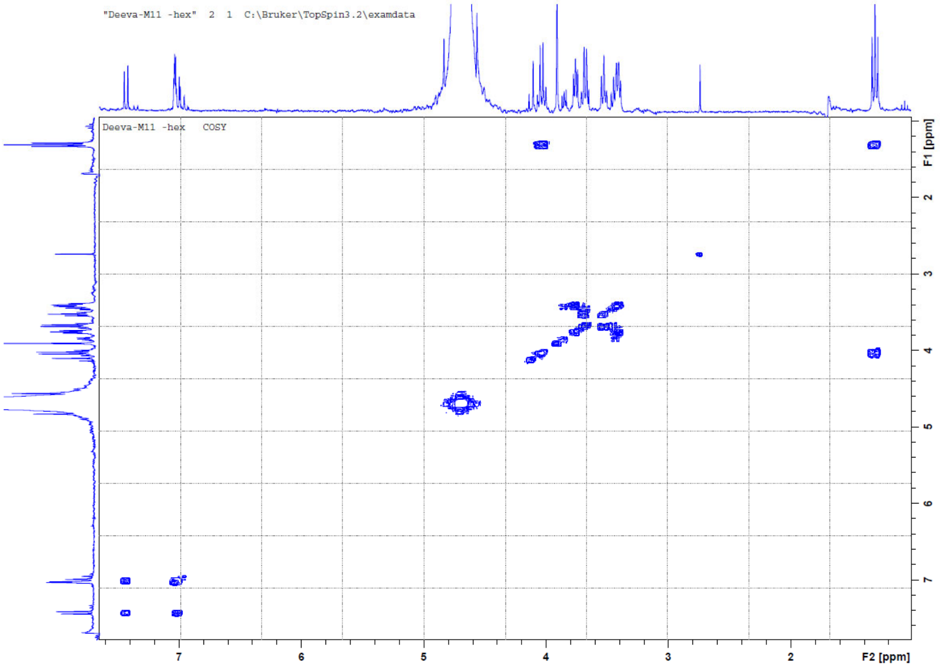
"Deeva-M11 -hex" 2 1 C:\Bruker\TopSpin3.2\examdata
Deeva-M11 -hex COSY
F1 [ppm]
F2 [ppm]

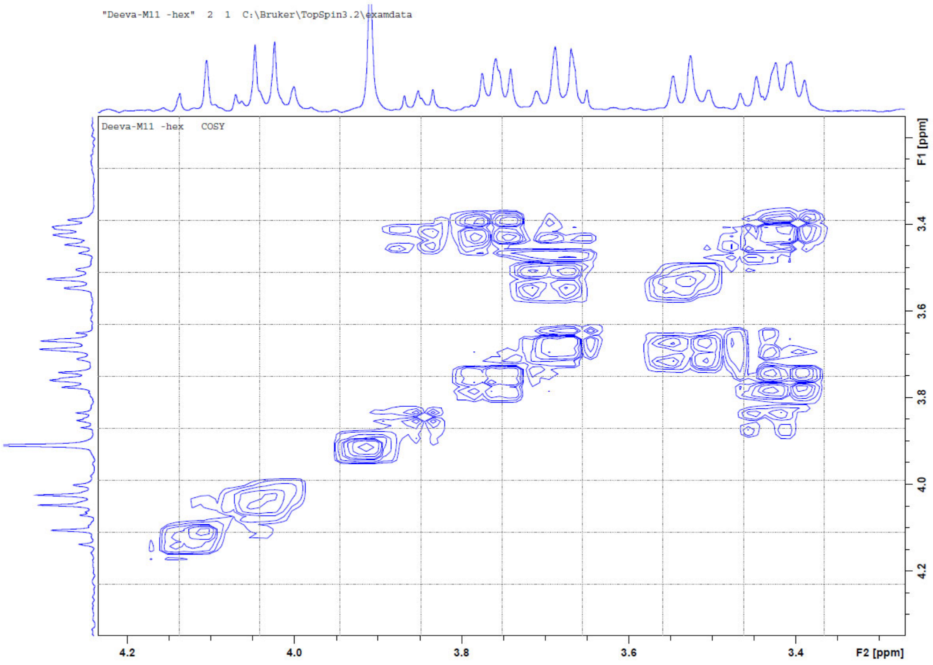

Supplementary Figure S4. ^{1}H-NMR and COSY NMR spectra of 2-[2-(3-oxomorpholine-4-yl)ethylthio]-5-ethoxybenzimidazole hydrochloride (M-11). Melting point of M-11 determined by capillary method using OptiMelt MPA 100 (Stanford Research Systems, USA) was 171-172 ^{0}C.
